# Hierarchically Structured Functionalized Polymer Monoliths Via Two-Photon Polymerization for Miniaturized HPLC Separation Columns

**DOI:** 10.1021/acs.analchem.6c02512

**Published:** 2026-07-22

**Authors:** Tobias Drieschner, Tobias Werres, Martin Klassen, Björn Lang, Anita Lorenz, Marc Brecht, Günter Lorenz, Thorsten Teutenberg, Andreas Kandelbauer

**Affiliations:** † Lehr- und Forschungszentrum Process Analysis and Technology (LFZ PA&T), 64332 Reutlingen University, Alteburgstrasse 150, 72762 Reutlingen, Germany; ‡ 271926 Institut für Umwelt & Energie, Technik & Analytik e. V. (IUTA), Bliersheimer Str. 58-60, 47229 Duisburg, Germany; § Institute of Physical and Theoretical Chemistry, University of Tübingen, Auf der Morgenstelle 18, 72076 Tübingen, Germany; ∥ Department of Natural Sciences and Sustainable Resources, Institute of Wood Technology and Renewable Materials, BOKU University, Konrad-Lorenz-Straße 24, 3430 Tulln an der Donau, Austria

## Abstract

Miniaturization of liquid chromatography (LC) systems offers numerous advantages. Nevertheless, miniaturized LC is still not widely used. Here, we present the additive manufacturing of hierarchically structured macro- and mesoporous functionalized polymer monoliths and their application for miniaturized reversed-phase chromatographic separations. A CAD-predesigned macroporous structure was generated by 3D printing of a repetitive design of Schoen gyroids from a photoresin via two-photon polymerization. For this, a photoresin formulation was developed containing a porogenic solvent to induce the formation of mesopores and a hydrophobic monomer to achieve a surface functionality suitable for reversed-phase chromatography. This strategy enables independent tuning of macropore geometry and mesoporosity, which is not readily achievable by conventional polymer monolith synthesis. Successful fabrication of hierarchically structured polymer monoliths was demonstrated by scanning electron microscopy and nano-computed tomography. The monolithic stationary phase was connected to a microfluidic system, and chromatographic separation of the nonpolar analytes naphthalene and fluoranthene was demonstrated. This highlights the applicability of the hierarchically structured porous functionalized polymer monolith as a reversed-phase stationary phase.

## Graphical abstract

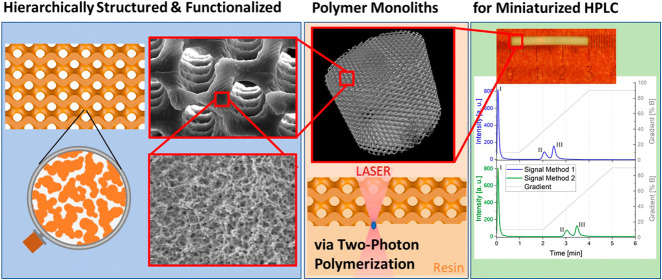

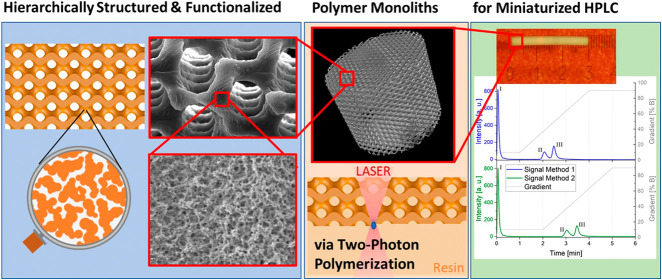

## Introduction

In response to the increasing demand for resource-efficient, rapid, and high-resolution analytical techniques, the miniaturization of chromatographic systems has become a central focus in analytical chemistry.^1–3^ Miniaturized high-performance liquid chromatography systems offer distinct advantages over conventional setups, including reduced sample and solvent consumption, faster analysis times,^4^ and lower operational costs. In addition, their compatibility with automated and portable platforms, such as lab-on-chip devices and point-of-care diagnostic tools, makes them particularly attractive for emerging applications in omics research, bioprocess monitoring,^5^ and on-site environmental analysis,^6^ where high throughput and sustainability are increasingly important. Traditionally, miniaturized HPLC columns are obtained from fused silica capillaries that are packed with porous silica particles via the slurry-packing method, with porous frits retaining the stationary phase. However, this approach is tedious and may result in inhomogeneously packed beds, leading to limited reproducibility, extensive quality control requirements, and high rejection rates if the column ID is below 1 mm. Chromatographic efficiency is further compromised by packing defects and wall effects, which lower the theoretical plate number. While reducing particle size can improve the separation efficiency, it also exponentially increases column backpressure, as described by the Darcy equation, thus imposing practical constraints on system design and operation. Another critical challenge in miniaturized LC systems is the impact of extra-column volume, including hardware components such as column frits, on band broadening and overall separation efficiency.^7^ Some of these disadvantages can be avoided by using fused silica monoliths as miniaturized chromatographic column materials which may be produced via sol–gel processing to form a stationary phase directly inside a fused silica capillary, thereby eliminating the packing procedure and all associated problems such as density gradients and the constraints of spherical particle shapes.

As an alternative to fused silica monoliths, many studies on monolithic stationary phases based on organic polymers were performed over the last few decades.^8–11^ Polymer-based monolithic columns^12^ consist of single continuous porous networks formed in situ, typically exhibiting interconnected flow-through macropores that ensure convective mass transport, alongside a finer micro- and mesoporous substructure.^13^ The macropores facilitate the flow of the mobile phase whereas smaller pores within the polymer skeleton increase the accessible interaction area. However, conventional polymer monoliths usually exhibit much lower specific surface areas compared to porous silica-based stationary phases.^14,15^ One particular advantage is the ability to synthesize the monolith directly in the intended mold, thereby omitting the crucial step of packing the stationary phase into the column housing. Numerous procedures were proposed and applied to generate polymer monoliths in capillaries,^8,16–18^ stainless steel or glass tubes,^18,19^ or chip-based flow channels.^20–24^ These monolithic columns typically exhibit low back pressure due to their large macropores and high porosity,^11,25^ compared to conventional columns packed with particles. Porous polymer monoliths have, for instance, been prepared directly in fused silica capillaries by UV-initiated photopolymerization reactions to yield strong cation exchanging stationary phases for separating peptides and proteins.^26,27^ However, their pore structures and macropore size distribution usually show a certain degree of randomness and inhomogeneities significantly impact band broadening while a uniform macropore network promises enhanced separation performance.^29,11,28^

However, by this method no predefined three-dimensional macro-porous structures of tailored geometries, strut widths and channel dimensions are accessible. A promising approach to generate such regular macroporous solids is using 3D printing of repetitive structural patterns. This technique allows for the digital design of structured solids, which can be tailored to the chromatographers’ needs. After a computer-generated design is created, it is subsequently fabricated using a suitable additive manufacturing process. Additive manufacturing of stationary phases enables fabrication of highly homogeneous and geometrically complex monolithic structures of defined morphology based on organic polymers.^29,30^ Hence, this approach was pursued in the past decade by numerous research groups.^31–42^

Since the first attempts by Fee et al.^31^ to fabricate stationary phases containing parallel channels, herringbone channels or octahedral arrays many strategies to print chromatographic columns were explored.^30,32–39^ Digital Light Processing (DLP) has proven an immensely powerful tool for this purpose allowing for printed microdevices with well-defined microchannels with diameters down to 18 μm x 20 μm.^23^ Wen et al.^38^ demonstrated the large scale printing of robust chromatographic columns for reversed phase (RP) and hydrophobic interaction (HIC) liquid chromatography using DLP. Directly printed monolithic ion exchange adsorbers were demonstrated.^33^ A review summarizing recent advances in the additive manufacturing of stationary phases was recently published by Conti and Dimartino.^40^

One particular aspect still under investigation is the reduction of feature size, which directly affects plate height and, consequently, separation performance. For example, Gritti and Nawada^34,35^ used a DLP printer equipped with photomasks to achieve features as small as 20 μm. However, for these small sizes, they reported a low precision of the printed channel dimensions (as much as 25% RSD in channel width) which leads to large dispersion effects and inevitable short-range interchannel velocity biases.^35^ In addition, the presented monoliths neither addressed mesoporosity nor surface functionality suitable for chromatographic purposes. These latter characteristics are known to be critical for broad chromatographic application, but impose further challenges on the manufacturing process in addition to further reducing the size of the printed features.

To achieve mesoporous structures in 3D printed monoliths, a widely used approach is adding porogenic solvents to the photoresins which induces phase separation during polymerization and thereby the formation of mesopores.^41,42^ Common solvents used as porogens are, for instance, butanol, cyclohexanol, dodecanol and mixtures thereof.^13,43^ During the reaction, nuclei of oligomers and polymers are formed and initially suspended in a liquid environment consisting of the porogenic solvent and unreacted monomers. As the monomer is consumed during further polymerization, the nuclei grow, agglomerate, and become cross-linked, leaving some space between the agglomerates which is occupied by the solvent thus forming a mesoporous network.^44^ By proper selection of the porogen (e.g., based on Hansen parameters), and both the monomer to porogen ratio, and the initiator type and concentration, it is possible to generate pore networks in the intermediate nanometer range. Thus, hierarchically structured polymer monoliths containing pores on different scales can be generated.

Conti et al.^36^ reported an approach to manufacture regular monolithic structures using DLP with a photocurable resin containing porogens and a bifunctional monomer. In their effort to explore the minimal feature size, they produced structures based on a triply periodic minimal surface (TPMS) Schoen gyroid with wall thicknesses of 300 μm, 200 μm and 150 μm. With structures of wall sizes below 200 μm, the authors reported frequently blocked pores and reproducibility problems with deviations in wall thicknesses of up to 80 %, which illustrates the challenges involved in reducing the feature size with this technique.

A technology suitable to surpass the limitations of DLP technology is two-photon polymerization (2PP), also known as direct laser writing (DLW). Among the various approaches of additive manufacturing, 2PP shows the highest resolution down to a few hundred nanometers.^45^ This technology enables reproducible generation of macroporous networks of defined geometry in the lower micrometer range suitable for chromatographic applications.

Scheme 1 shows how such macroporous structures are printed by 2PP of a digitally designed 3D model and how the mesoporous structures are formed during polymerization-induced phase separation. In addition, the surface characteristics of the monolith can be optimized for chromatographic application by adding a monomer carrying a suitable functional group for optimal interaction with analyte molecules.^10,42^

**Scheme 1:**
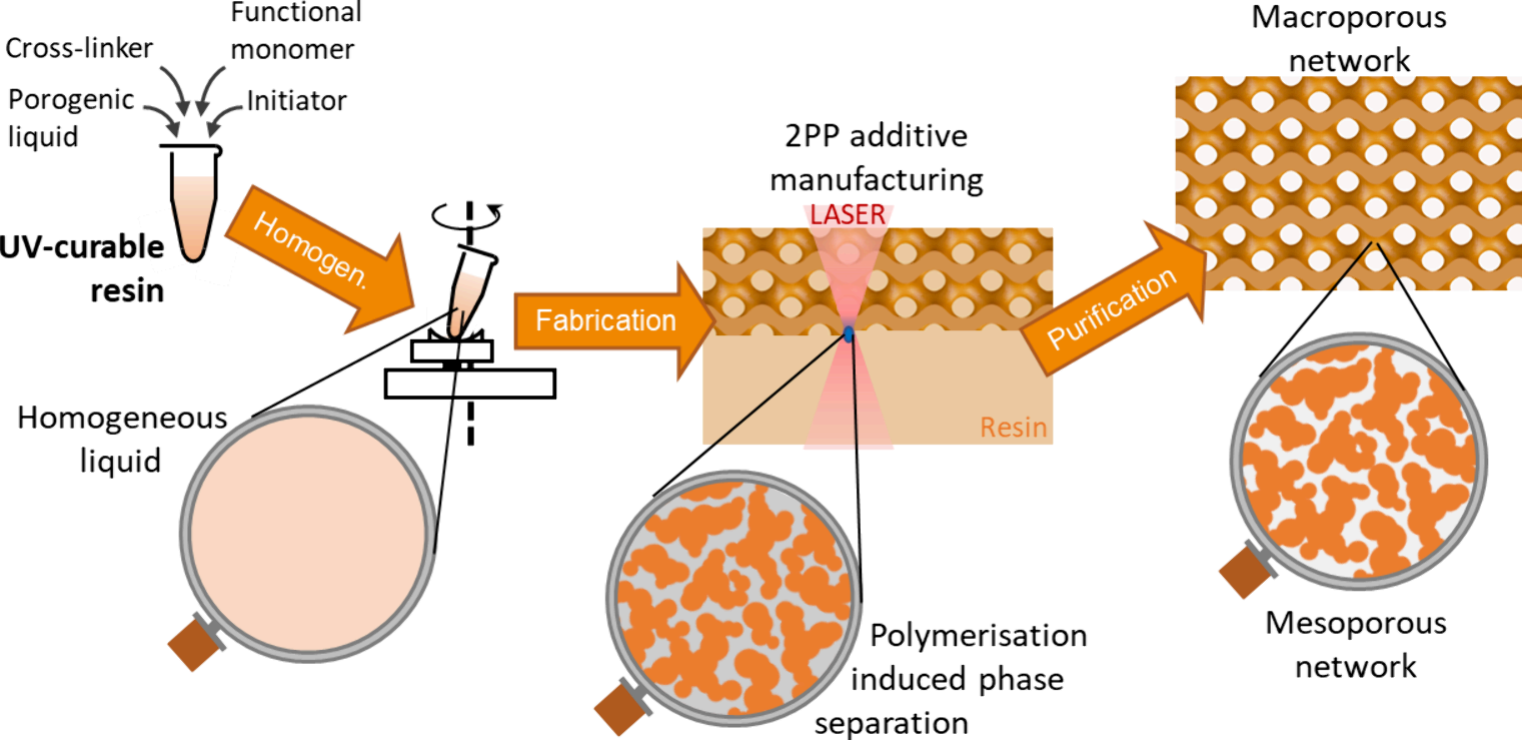
Schematic Depiction of Additive Manufacturing of Hierarchically Structured Porous Polymer Monoliths^a^

a A photoresin consisting of crosslinking monomer, functional monomer, porogenic solvent, and photoinitiator is used to manufacture the digitally predesigned macroporous structure by 2PP while simultaneously a mesoporous structure is formed by polymerization-induced phase separation. By removing non-reacted resin, the hierarchical porous polymer monolith is accessible for chromatographic application.

The applicability of 2PP for manufacturing chromatographic supports was first reported by Matheuse et al.^32,45^ who explored the minimum feature size possible with 2PP. Due to the used high resolution, they observed very long printing times and concluded that 2PP would be not a feasible technique to produce monolithic columns. In the present study, we accelerate the printing process by a factor of 50 via lower resolution. A detailed comparison between their printing parameters and ours is given in SI. Considering the technological progress in developing commercial 2PP printers, a substantial general increase in manufacturing speeds can be noted during the past years. Based on analyzing print rates used in scientific studies published between 2007 and 2025, a recent study^46^ has shown that the printing speed of 2PP systems increased by a factor of approximately 2 per year.

While many formulations were suggested for photocurable resins with which monolithic chromatographic columns were prepared (e.g.,^12,13,25^ ^,^ ^40,43,47,48^ ), the 2PP printing process imposes special requirements regarding viscosity, refractive index and volatility on the photoresin. Hence, especially when mesoporous polymeric monoliths are addressed with 2PP new recipes are required and need to be developed. By including a functional monomer in the photoresin formulation, an intrinsically functionalized monolith can be achieved during photopolymerization and no need for subsequent functionalization arises.

Here, we report the 2PP manufacturing of hierarchically structured macro- and mesoporous polymer monoliths based on a newly developed functionalized photocurable acrylic resin suitable for reversed phase chromatography. A distinctive advantage of the presented approach is the decoupling of macropore architecture from mesopore formation: the macroporous geometry is defined by CAD and realized by 2PP, while mesoporosity is independently adjusted through the photoresin chemistry.

## Results and Discussion

### Preparation and Characterization of Additively Manufactured, Hierarchically Structured Polymer Monoliths

The macroporous network was designed by periodic repetition of a Schoen gyroid-based element (Figure 1A). The Schoen gyroid^49^ is a triply periodic nonself-intersecting surface with minimized local surface area^50^ producing structurally strong periodic packing with interconnected flow paths with minimal flow resistance.^29,51^ It is a bionic structural motif in form of a labyrinthine 3D network with curved surfaces repeating in all directions. It is widely used in additive manufacturing^52^ because the mathematical representation of its structure can readily be translated into a 3D-printing process. As a special form of a triply periodic minimal surface (TPMS) structure, the gyroid is characterized by uniform strength, good fluid flow properties and no abrupt changes in direction, which is particularly advantageous for HPLC and makes processing of such structures interesting from a technological perspective. It was shown to uniformly distribute internal stresses in a controlled manner. Thereby, local stress overloads are avoided minimizing the risk of crack formation and propagation that could cause mechanical destruction and loss of function of the stationary phase.^29,51,53–55^ In computational studies, TPMS structures were shown to have smaller HETPs compared to spherical particle arrangements due to their highly uniform flow channels.^56^ However, a detailed investigation on the separation behavior of various TPMS structures^57^ showed that no single geometric or fluid-dynamic parameter can be identified that would explain the hydrodynamic performance of such structures and that the dispersion coefficients would have to be analyzed on a case-to-case-basis. The gyroid structural motif was used in 3D-printing of chromatographic columns earlier with promising results. For instance, a patent^58^ described gyroidal column materials based on chemically modified agarose and cellulose hydrogels for separating biomolecules by ion exchange chromatography. The suitability of Schoen gyroids for stationary phases manufactured by DLP was also shown previously by Simon et al.^33^ and Conti et al.^36^

**Figure 1:**
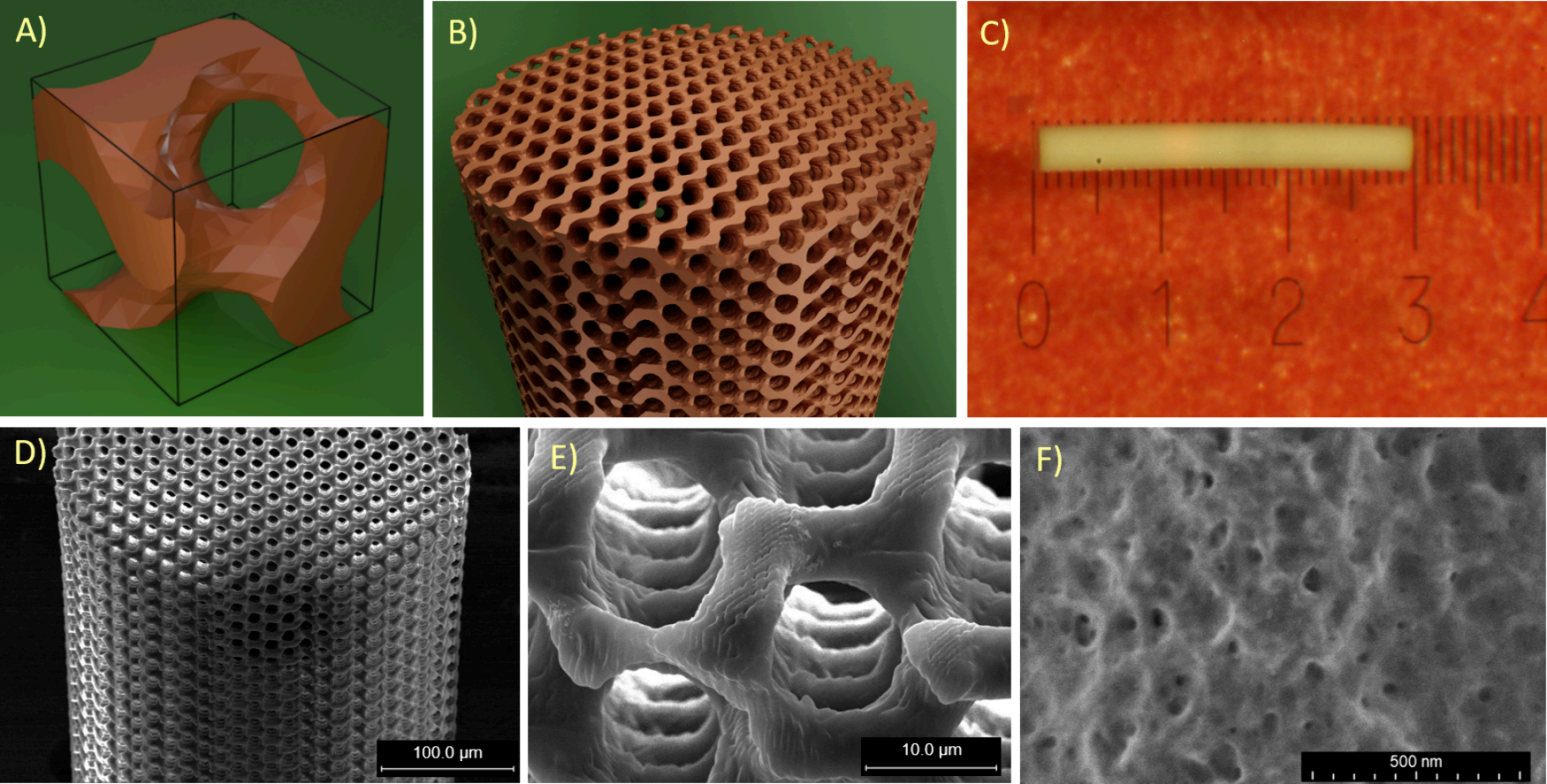
A) 3D-model of one single gyroid element; B) upper end of a cylindrical structure designed by consecutive iteration of the gyroid element resulting in a regular macroporous network. C) microscopic image of an additively manufactured monolith with total length of 3 mm and diameter of 350 μm. D-F) SEM images of polymer monolith at different resolutions to highlight the macropores (D, E) and the mesopores (F) of the material.

In our study, cylindrical monoliths of 3 mm length and a diameter of 350 μm were manufactured using two-photon polymerization (2PP). The monoliths consisted of a macroporous network generated by regular iteration of gyroid elemental cells (Figure 1A) with an edge length of 20 μm (indicated by the black cube in Figure 1A). This design inherently involves parallel and interconnected channels along the flow direction with a channel diameter of 7.5 μm. The strut size was 6.2 μm. A macroporosity of 70% was chosen because of reported favorable permeability properties at such a void volume fraction.^56^ TPMS structures were shown in theoretical studies to have higher permeability than spherically packed columns at porosities of around 70% whereas at higher porosities, the Darcy number decreases for TPMS monoliths due to an increase in hydraulic diameter.^56^ Further information on structural dimensions of the polymer monolith and relevance for chromatographic separation is given in SI.

A 3D-rendered model of the macroporous network is given in Figure 1B. The photoresin used was poly­(meth)­acrylate cross-linked with pentaerytrithol tetra-acrylate (PETA) which yielded a rigid polymer monolith. A small amount (5% w/w) of dodecyl methacrylate (DMA) as a functional monomer was used in the resin formulation to obtain nonpolar monolith surfaces. Mesopores were introduced during 2PP using butyl benzoate as a porogenic solvent causing phase separation during polymerization.

Figure 1C shows a typical polymer monolith produced with 2PP after removal of unreacted resin (see SI). The microstructure of the monoliths was analyzed by SEM (Figure 1D-F). Figure 1D shows the overall macroporous network structure comparing well to the digital 3D design shown in Figure 1B. Figure 1E shows a smaller section of the macroporous structure. The stripes visible on the monolith surface originate from the layer-by-layer and line-by-line process of additive manufacturing.^36^ Figure 1F shows the monolith surface in higher resolution, highlighting the mesoporous nature of the material. The size of the mesopores was in the range from 10 to 100 nm as estimated from SEM micrographs semiquantitatively. This mesoporous structure increases the accessible surface area of the printed polymer skeleton and resulted from including butyl benzoate as a porogen in the photoresin formulation which is illustrated by the SEM micrographs shown in Figure S1 in the SI.

X-ray nano-computed tomography (Nano-CT) was used to examine the structural integrity and how well the macroporous morphology of the fabricated polymer monoliths matched the original digital 3D design. X-ray nano-CT should reveal whether unreacted resin or other impurities were still attached after the cleaning process and whether geometry of the column, dimensions and porosity matched the CAD. Figure 2 shows the 3D reconstruction of a segment of a monolithic column.

**Figure 2:**
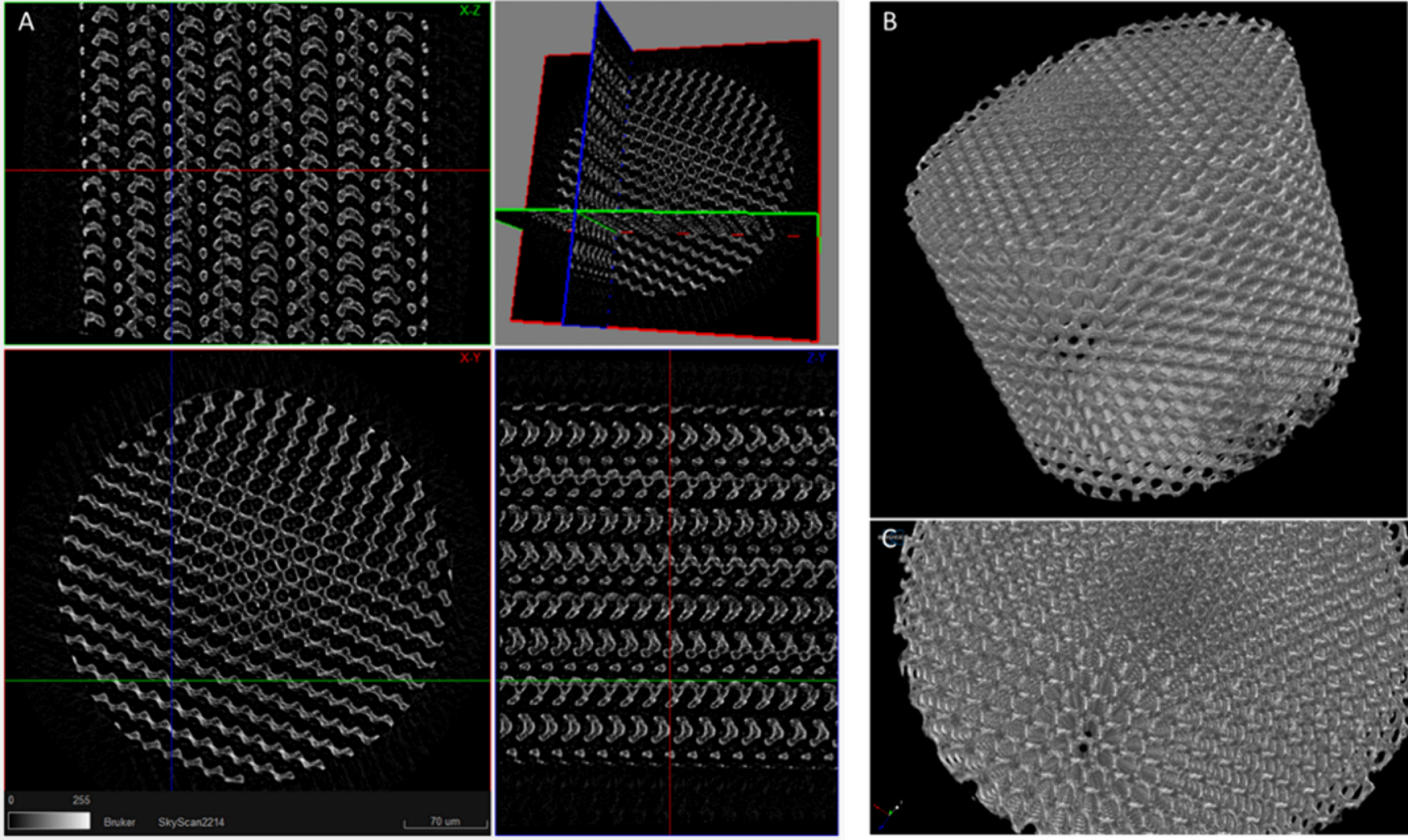
Nano-CT analysis of internal structural integrity and homogeneity of a polymer monolithic column with a macroporous network of gyroid elements: A: Example of cross-sectional images after reconstruction in the x/z, x/y and z/y planes, B and C: 3D renderings from different perspectives into macroporous channels.

Cross-sectional views in all three spatial directions revealed that there was no residual photoresist retained in the macropores after washing the monolith thoroughly (see SI). Figure 2 also shows that the printed structures correspond well to the specifications defined in CAD. The nano-CT scans show no signs of cracks, air bubbles or other defects present in the monolith. Analysis of the reconstructed nano-computed tomograms revealed a porosity of 71%, corresponding well to the theoretical value of 70% calculated for the gyroid structure (see SI). Comparison of the dimensions: Column diameter according to CAD: 350 μm, according to nano-CT analysis: 335 μm. This shows that the material used for 3D printing exhibits only slight shrinkage (ca. 4%). Furthermore, the dimensions determined using nano-CT agree well with the SEM results. Although examination using SEM provides a significantly higher lateral resolution, only the outer surface of the monolith can be explored by SEM whereas nano-CT allows investigating the inner volume of the monolith.

Nano-CT was not used to characterize the mesoporous structure of our monoliths. Although in earlier studies, nano-CT analysis was successfully employed to also investigate the pore structure and pore size distribution of 3D-printed separation materials,^59^ the mesopore sizes in our monoliths are too small (between 10 and 100 nm) for nano-CT analysis.

### Use of Additively Manufactured Polymer Monoliths in Chromatographic Separation

To evaluate the applicability of the novel monoliths in chromatographic separation, a chromatographic column was built by implementing two monoliths each of 3 mm length in a fluorinated ethylene propylene (FEP) sleeve (see SI). The sequentially arranged monoliths were tightly connected between two fused silica capillaries inserted in the sleeve from both ends and precisely positioned at the ends of the monoliths. These capillaries were used to connect the monolithic column to the μ-LC system comprising an autosampler, gradient pumps and a UV-detector. A gradient elution experiment was performed using a test mixture consisting of three aromatic analyte compounds: the polar acetylsalicylic acid (ASA) and two nonpolar polycyclic aromatic hydrocarbons naphthalene and fluoranthene. All separations were conducted using the hierarchically structured polymer monoliths functionalized with dodecyl methacrylate to impart reversed-phase properties. To verify successful functionalization, an initial isocratic hold was incorporated into the gradient program. The resulting chromatograms, averaged from triplicate measurements, are shown in Figure 3.

**Figure 3:**
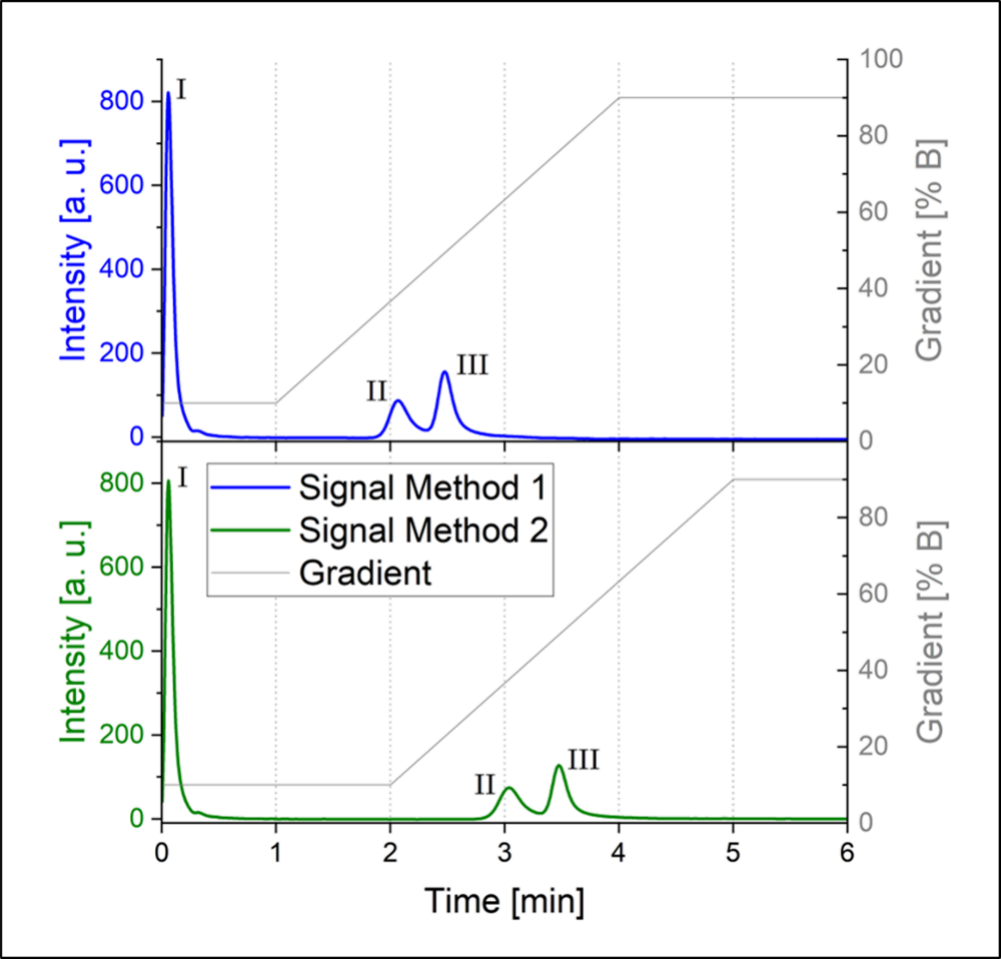
Chromatograms of the test mixture separated on the additively manufactured hierarchical monolith using two gradient methods differing only in the duration of the initial isocratic hold. Chromatographic conditions: Flow rate = 20 μL/min; injection volume = 50 nL; column dimensions = 6.0 × 0.35 mm (length x inner diameter). Analyte assignment: I = acetylsalicylic acid, II = naphthalene, III = fluoranthene.

As shown in the chromatogram of Figure 3, the three components are separated within a 3 min gradient. Due to its high polarity, ASA eluted close to the system void time (0.06 min), while the nonpolar analytes naphthalene and fluoranthene exhibited retained elution at 2.07 min and 2.48 min, respectively, with a chromatographic resolution of 0.54 for the critical peak pair. Although baseline separation was not achieved, the low resolution is primarily due to pronounced tailing of the analyte peaks. It should be emphasized that the observed tailing is not intrinsic to the monolithic stationary phase. Based on system dead-volume, a substantial contribution from extra-column band broadening is expected. In particular, dispersion may arise from downstream capillary connections, detector interfaces, and potential mismatches at column-capillary junctions (see also SI). This retention behavior confirms successful surface modification of the monolith, as the nonpolar analytes interacted strongly with the functionalized stationary phase and were only eluted upon increasing the mobile phase organic modifier concentration.

To further validate the interaction of analytes and functionalized stationary phase, a second experiment was conducted with a 1 min extension of the initial isocratic hold (lower chromatogram, Figure 3). While ASA’s elution time remained unchanged, the retention times of naphthalene and fluoranthene shifted to 3.04 min and 3.48 min, respectively, with a reduced resolution of 0.50. The decrease in resolution is attributed to band broadening occurring during the extended isocratic plateau, where the analytes remain associated with the stationary phase. More specifically, surface diffusion along the stationary phase can contribute to band broadening over time, even when the analytes are retained at the column inlet.

However, a substantial fraction of the observed peak tailing may originate from extra-column band dispersion rather than solely from the monolithic structure. In particular, imperfect alignment and coupling of the two sequential monolithic segments, as well as dispersion effects arising from downstream capillary and detector volumes. This demonstrates that naphthalene and fluoranthene are retained under aqueous conditions and require a mobile phase polarity shift for elution. In contrast, ASA’s elution profile remains unaffected, confirming negligible interaction with the hydrophobic stationary phase. The monolithic column used in this proof-of-concept study exhibits a relatively low binding capacity. Partial residence of analytes in the mobile phase may additionally contribute to band broadening during the isocratic hold. Based on system volume considerations, extra-column contributions are further supported quantitatively. Using the measured flow rate (20 μL/min) and the retention time of a nonretained tracer (0.06 min), the total system volume is estimated to be approximately 1.2 μL. In comparison, the geometrically estimated column volume (D^2^π/4·L·porosity) is approximately 0.4 μL, indicating that a substantial fraction (∼0.8 μL) of the system volume is located outside the stationary phase. This confirms that extra-column dispersion represents a significant contribution to overall band broadening in the current setup.

The authors acknowledge that the present chromatographic performance is suboptimal and does not yet reflect the theoretical performance limits for TPMS stationary phases (see comments in SI). Future iterations will focus on (i) reducing intrinsic system dead volume by employing low-volume μ-LC hardware and detection systems, (ii) improving dead-volume-free coupling between printed monoliths and fluidic interfaces, and (iii) increasing column length to reduce the relative contribution of extra-column dispersion. Future work will also investigate alternative TPMS geometries and smaller feature sizes.

Overall, these findings support the successful functionalization of the monolith and its suitability for reversed-phase chromatographic separations.

## Conclusions

The additive manufacturing of hierarchically structured macro- and mesoporous polymer monoliths for use as stationary phases in miniaturized reversed-phase liquid chromatography was investigated. A macroporous monolith, generated by periodic repetition of a Schoen gyroid-based cubic cell, was fabricated via 2PP. Concurrently, mesopores were generated independently of the printed macropore architecture via polymerization-induced phase separation, using butyl benzoate as porogenic solvent in the photoresin. Incorporation of dodecyl methacrylate introduced nonpolar surface functionalities suitable for reversed-phase interactions. The hierarchical structure was verified by SEM, confirming the intended macroporous and mesoporous morphology. Nano-CT analysis of macropores demonstrated defect-free fabrication and effective removal of nonreacted resin. To integrate the monoliths into fluidic systems with minimal void volume, a simple assembly using an FEP sleeve and fused silica capillaries was developed. This setup also allowed serial connection of multiple monoliths to extend stationary phase length. Chromatographic tests confirmed reversed-phase mechanism: nonpolar analytes (naphthalene, fluoranthene) were retained and eluted in accordance with gradient changes, while the polar compound acetylsalicylic acid eluted at the void time. These results demonstrate the suitability of the novel type of additively manufactured hierarchical polymer monoliths for miniaturized liquid chromatography. Although current fabrication (3 mm × 350 μm) requires about 2 h, ongoing advances in two-photon polymerization promise substantial acceleration. Future prospects include direct in situ fabrication within transparent microfluidic systems and enabling digital design of tailored macroporous geometries for optimized chromatographic performance.

## Supplementary Material



## Notes

Materials and methods: chemicals and reactants, formulation of the photoresin, design and manufacturing of polymer monoliths, characterization of polymer monoliths, microfluidic integration and chromatographic experiments (PDF)

## Notes

This article is dedicated to the memory of Günter Lorenz.
