# Supplementary material for: Hierarchically Structured Functionalized Polymer Monoliths Via Two-Photon Polymerization for Miniaturized HPLC Separation Columns
Source: Anal Chem. 2026 Jul 22;98(30):22009–16. doi: 10.1021/acs.analchem.6c02512 (PMC13449221; doi:10.1021/acs.analchem.6c02512)
Supplement: Supplementary file 1 [file ac6c02512_si_004.pdf]

Supporting Information to: Hierarchically Structured Functionalized Polymer Monoliths via Two-Photon Polymerization for Miniaturized HPLC Separation Columns

Tobias Drieschner[1], Tobias Werres[2], Martin Klassen[2], Björn Lang[2], Anita Lorenz[1],Marc Brecht[1,3], Günter Lorenz[1], Thorsten Teutenberg[2] and Andreas Kandelbauer,[1,4]*

[1]Lehr- und Forschungszentrum Process Analysis and Technology (LFZ PA&T), Reutlingen University, Alteburgstrasse 150, 72762 Reutlingen, Germany

[2]Institut für Umwelt & Energie, Technik & Analytik e. V. (IUTA), Bliersheimer Str. 58-60, 47229 Duisburg, Germany

[3]Institute of Physical and Theoretical Chemistry, University of Tübingen, Auf der Morgenstelle 18, 72076 Tübingen, Germany

[4]Department of Natural Sciences and Sustainable Resources, Institute of Wood Technology and Renewable Materials, BOKU University, Konrad-Lorenz-Straße 24, 3430 Tulln an der Donau, Austria

Table of Contents

Chemicals and Reactants (p. S3)

Formulation of the Photoresin (p. S3)

Design and Manufacturing of Polymer Monoliths (p. S4)

Comment on Structural Dimensions of the Polymer Monolith and Relevance for Chromatographic Separation (p. S6)

Characterization of Polymer Monoliths (p. S7)

Effect of the Porogenic Solvent on Mesopore Formation(p. S8)

Microfluidic Integration and Chromatographic Experiments (p. S9)

Comment on Wall channeling Effects (p. S11)

Comment on Chromatographic Performance and Technical Limitations of the System Presented (p. S11)

References

Chemicals and Reactants

Pentaerytrithol tetra-acrylate (PETA) and 4,4'-bis(diethylamino) benzophenone were purchased from Sigma-Aldrich Chemie GmbH (Steinheim, Germany). Dodecyl methacrylate (DMA) was obtained from Fluorochem (Hadfield, UK), and butyl benzoate from BLD Pharmatech GmbH (Reinbek, Germany). 2-propanol (HPLC-grade) and propylene glycol methyl ether acetate (PGMEA, ≥ 99 % purity) were purchased from Carl Roth GmbH + Co. KG (Karlsruhe, Germany). All chemicals were used as received.

For the chromatographic experiments LC-MS grade acetonitrile (ACN) purchased from Th. Geyer (Chemsolute, Renningen, Germany) and water supplied by a PURELAB Chorus 1 from ELGA LabWater (Lane End, England) were used. To adjust the pH, formic acid (Sigma-Aldrich, Seelze, Germany) was added to a total volume concentration of 0.1 % to the mobile phase solvents. Acetylsalicylic acid (≥ 99 %), fluoranthene (≥ 99 %), and naphthalene (≥ 99 %) were purchased from Sigma-Aldrich (Seelze, Germany).

Formulation of the Photoresin

The photoresin for the hierarchically structured monoliths was prepared by mixing the cross-linking monomer PETA and DMA as a functional monomer in mass fractions of 95 % and 5 %, respectively. To this monomer mixture, the porogenic solvent butyl benzoate was added in a mass ratio of 70 % monomer mixture and 30 % butyl benzoate. In addition, 1.5 % (w/w(monomers)) of the initiator 4,4'-bis(diethylamino) benzophenone was added. The resin was kept in a polystyrene tube covered with aluminium foil, mixed intensively, and allowed to settle for at least 24 hours before further use.

In order to examine the effect of the porogen on pore formation, the following recipes were used for cubic test structures: a) porogen-free formulation consisting of PETA + 1,5 % (w/w(monomer)) of the photoinitiator 4,4'-bis(diethylamino) benzophenone (for the material shown in Figure 2 A, C); it was necessary to omit the addition of 5% DMA from this

formulation, as the two monomers do not mix without the addition of the pore-forming solvent. b) porogen-containing formulation consisting of 95 % PETA + 5 % DMA. To this monomer mixture, the porogenic solvent butyl benzoate was added in a mass ratio of 70 % monomer mixture and 30 % butyl benzoate. In addition, 1.5 % (w/w(monomers)) of the initiator 4,4'-bis(diethylamino) benzophenone was added (for the material shown in Figure 2B, D).

Design and Manufacturing of Polymer Monoliths

A cubic 3D-mesh consisting of a gyroid triply periodic minimal surface was generated using a MATLAB script according to the following shape equation (eq. 1)[1]:

(eq. 1) $\sin x \cos y + \sin y \cos z + \sin z \cos x = c$

where x, y, and z are the cartesian coordinates and c means a volume-fraction defining constant. A gyroid divides space into two interconnected compartments (solid material and void space) and by adjusting the constant c, the volume fractions of the two compartments can be defined. In the present study, c was set to 0.6, and the edge length of the cubic elemental cell was defined as 20 µm. The mesh was imported into the software package OpenSCAD version 2021.01. A dense 2D-array in the x- and y-space was created by regular iteration. The array was limited to a cylindrical top-level geometry (height of the cylinder was set in the z-dimension) to yield a diameter of 350 µm. In the resulting 3D design, the smaller compartment was defined as the solid phase, while the larger compartment remained void. Void space representing the macroporosity of the monolith can be calculated from the constant c according to equation 2 (eq. 2[1])

(eq. 2) $V_f = 0.5 - 0.333c$

where V_f is the volume fraction of the solid compartment. For c = 0.6, the structure consisted of 30 % solid and 70 % macropores.

The intended height in the z-space of 3 mm was achieved in the consecutive fabrication process by programming the printer to stack the required number of x,y-arrays on top of each other. This was done by importing an STL-file with the 3D model of a single x,y-array in the device-specific software package Describe version 2.7. The fabrication settings were set to a scan speed of 85,000 µm/s and 100 % laser power.

A Photonic Professional GT printer (Nanoscribe GmbH & Co KG, Eggenstein-Leopoldshafen, Germany) equipped with a 25x magnification objective (Carl Zeiss Microscopy Deutschland GmbH, Oberkochen, Germany) was used for fabrication. The device-specific control software NanoWrite version 1.10.6 was used. Polymer monoliths were fabricated using the photoresin on indium tin oxide coated-glass substrates (Nanoscribe GmbH & Co KG, Eggenstein-Leopoldshafen, Germany) in dip-in laser lithography mode. The objective was covered with cling film according to a procedure described by Toulouse et al.[2]. After fabrication, remaining excessive amounts of resin were removed by submerging the monolith in PGMEA and iso-propanol for 30 min and 2 min, respectively. Washing with PGMEA and isopropanol dissolves unpolymerized monomer as well as porogenic solvent and removes them from the crosslinked monolithic structure. Afterwards, the monoliths were dried and store until further usage.

The printing conditions used here allow for sufficient details in structural features and satisfactory printing times. It has been reported in the literature that 2PP would not be a competitive technique for manufacturing 3D-printed monolithic chromatographic stationary phases. This is correct as long as very high demands with respect to structural details and resolution are aimed at. Matheuse et. al.[3] explored the minimum feature size possible with 2PP was explored by using high-resolution optics. By using a high-magnification (63x) large-numerical aperture (NA = 1.4) microscope objective, they managed to print pillar structures even smaller than 1 µm. Using the highest possible resolution resulted in comparatively long printing times for the production of very small structures. This of course is a disadvantage of

high-resolution 2PP and led the authors to conclude that 2PP was not ready yet for replacing the existing column fabrication methods. However, if not the highest resolution in structural features is intended or required, 2PP presents an interesting alternative 3D printing technology. For instance, if a cylindrical body with a length of 3 mm and a diameter of 350 µm is printed using a lower magnification objective (25x, instead of a 63x objective) and medium resolution parameters (slicing distance 1000 nm, hatching distance 500 nm, scan speed 85 mms-1, as used in the present study) instead of high resolution ones (slicing distance 200 nm, hatching distance 150 nm, scan speed 50 mms-1, as used by Matheuse et al. 2022[3] the required printing time can be reduced by a factor of about 50.

Comment on Structural Dimensions of the Polymer Monolith and Relevance for Chromatographic Separation

From a chromatographic perspective, the selected structural dimensions were chosen to match the characteristic length scales typically employed in micro- and nano-LC stationary phases. The 7.5 µm flow channels are comparable to the interstitial dimensions encountered in columns packed with particles and provide a compromise between permeability and mass-transfer efficiency.[4,5] At the same time, the mesoporous structure (10–100 nm) increases the accessible surface area available for analyte interaction and retention, while the ordered macroporous network ensures convective transport through the column. The hierarchical separation of transport and interaction length scales is a key design principle of monolithic stationary phases.[6–8] The selected gyroid unit-cell size (20 µm) and porosity (70 %) therefore place the structure within the dimensional regime generally considered suitable for miniaturized LC systems, where low hydraulic resistance, efficient mass transport, and reduced eddy dispersion are required simultaneously.[4,5,9,10] While further optimization of channel dimensions and column length will be necessary to maximize chromatographic

efficiency, the present dimensions were selected as a practical starting point for demonstrating the feasibility of additively manufactured hierarchically structured stationary phases.

Characterization of Polymer Monoliths

The microstructure of the polymer monoliths was observed using scanning electron microscopy (SEM) and nano-computer tomography (Nano-CT). For SEM imaging a Coxem EM-30^{N} (COXEM, Daejeon, Republic of Korea) equipped with a tungsten filament operated at 15 kV voltage and a secondary electron detector was used for analysis of the macroporous structure. For visualization of mesopores a Hitachi SU8030 instrument was applied. For SEM analysis with the COXEM device, the samples were sputtered with gold. SEM investigation using the Hitachi device was done without prior sputtering. For high-resolution SEM, cubic test structures were printed in order to generate flat surfaces whose surface properties can be satisfactorily characterized. On curved gyroid surfaces, it would have been difficult to create comparable surface segments.

For visualization of the inner morphology by Nano-CT monolithic columns were prepared on a small sample holder. They were subjected to nano-tomographic scanning using a maximum-resolution Nano-CT (SkyScan 2214, Bruker, Kontich, Belgium). The samples were rotated by 0.2 degrees in specific increments to scan 360 degrees across the vertical axis.

The voltage for the x-ray source was set at 40 kV and a current of 150 µA was used. A tungsten filament was used for electron beam generation, the focal spot size of which is specified at 800 nm (according to the manufacturer). During the imaging process, each degree of rotation was maintained for 9655 ms, using a sCMOS x-ray detector with 5056 x 2470 pixels. The image pixel size during scanning was 0.1 µm. An average of 3 frames was calculated per projection. The total scan time per sample was approximately 20 h.

Reconstruction was performed with NRecon Software (Version 2.2.0.6). Prior to reconstruction, slight (thermal) drifts and misalignment of the samples were corrected. The

corrected images were reconstituted using a correction of the ring artifacts of 5, and a correction of beam hardening of 20 %. For 3D rendering the software CTVox, Version 3.3.1, was used, cross sectional images were generated using DataViewer Version 1.8.0.1. Data Analysis (e.g. porosity) was performed using CTAn Version 1.23.0.2+. All Nano-CT software is from Bruker, Kontich, Belgium.

Effect of the Porogenic Solvent on Mesopore Formation

Figure S1 shows the effect of the porogenic solvent on mesopore formation during the 2PP process.

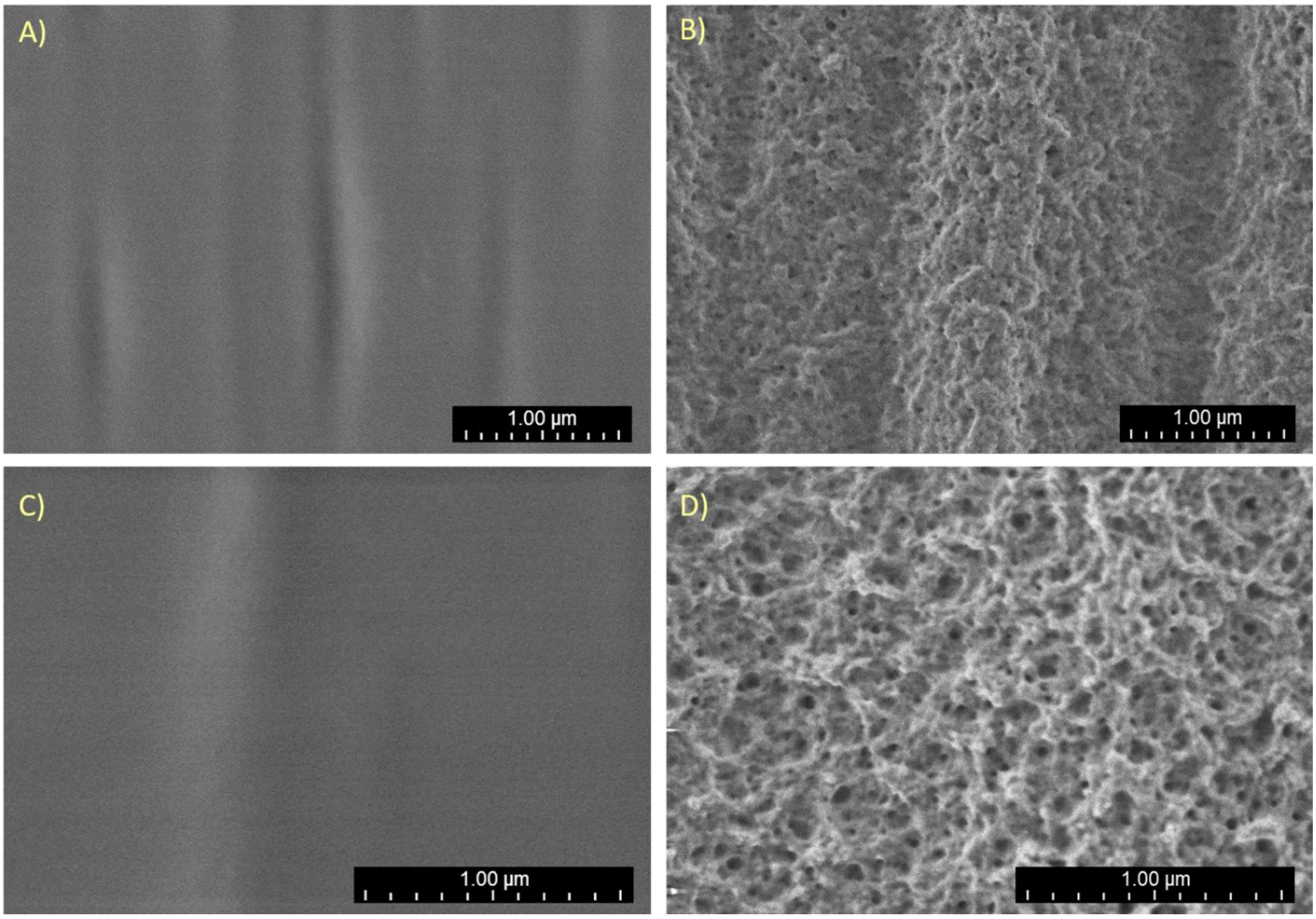

Figure S1. SEM images of test structures printed with porogen-free (A, C) and porogen-containing photoresin (B, D) at different magnifications.

Figure S1 compares the porosity of test structures printed by 2PP using the photoresin without porogen (Figures S1 A, C) and containing the porogen (Figure S1 B, D). In the SEM-images of the monoliths produced without a porogenic solvent, only the strip-like structures resulting from the printing process (vertically oriented in the Figure S1 A) are visible. Apart from that, the surface is very smooth. In the case of monoliths produced using porogens, the structure resulting from the printing process is overlaid by a rough, mesoporous texture.

Microfluidic Integration and Chromatographic Experiments

For integration into fluidic systems the manufactured polymer monoliths were inserted into fluorinated ethylene propylene (FEP)-sleeves (Part. Nr. 220564, BGB Analytik AG, Rheinfelden, Germany). When subsequently assembling the microfluidic system, the properties of mechanical FEP are exploited, particularly its low friction coefficient and mechanical flexibility. The FEP sleeve used is specified for capillaries with outer diameters of 275–315 µm. Two fused silica capillaries, each with an outer diameter of 365 µm and an inner diameter of 50 µm (Postnova Analytics GmbH, Landsberg, Germany), were inserted from opposite ends of the sleeve and precisely positioned at both ends of the monolith. When inserted into the sleeve, the fused silica capillaries expand the FEP sleeve locally, thereby creating a liquid-tight seal. In combination with the shrinkage of the printed monolith (approximately 4% as described in the manuscript), the monolith could be inserted into the sleeve with slight mechanical pressure. Using this approach, leak-free connections could be achieved up to approximately 100 bar system pressure. No additional bonding or chemical treatment steps were applied, the connection was achieved solely by mechanical assembly of the individual components.

All chromatographic experiments were conducted with an Ekspert nano-LC System (Sciex, Dublin, CA, USA), a micro-HPLC system consisting of an LC 400 autosampler for sample

injection and an LC 425 pump module. For data acquisition a 1260 Infinity II diode array detector WR (Agilent Technologies, California, USA) with a cell volume of 80 nL was used.

As a sample, a mixture of acetylsalicylic acid (0.05 g/L), fluoranthene (0.005 g/L), and naphthalene (0.0005 g/L) were dissolved in demineralized water with 0.6 % (v/v) acetonitrile. As the stationary phase two monoliths with a length of 3 mm each were inserted into one FEP sleeve fastened by capillaries. A void volume-free connection was confirmed using light microscopy. Thus, an effective chromatographic bed length of 6 mm was obtained. Demineralized water (reservoir A) and acetonitrile (reservoir B), both with 0.1 % (v/v) formic acid, were used as the mobile phase. The flow rate was set to 20 µL/min. By means of metered injection, a volume of 50 nL was transferred to the column. The separation was achieved according to the following gradient method: an initial isocratic step over 1 min with 10 % B is followed by a gradient to 90 % B over 3 min, and a final isocratic elution step over 2 min. The UV detection wavelength was set to 218 nm. The measurements were conducted in triplicates and the mean was calculated for further data handling. Furthermore, a blank run was conducted, in which 50 nL of demineralized water was injected. The resulting chromatogram was subtracted from the actual measurement to minimize baseline drift. To verify whether the intended surface-functionalization of the monoliths was achieved, the initial isocratic plateau was extended to two minutes in a further experiment. The slope of the gradient was kept constant.

The resolution R was calculated using the peak widths (w_h) at the full width at half maximum (FWHM) using the following equation:

$$R = 1.18 * \left(\frac{t_{r2} - t_{r1}}{w_{h1} + w_{h2}}\right)$$

where t_{r1} is the retention time of the first and t_{r2} the retention time of the second component.

Comment on Wall Channeling Effects

An additional potential source of dispersion is wall-channeling at the interface between the monolith and the FEP sleeve. Although such effects cannot be completely excluded in the present proof-of-concept assembly, nano-CT analysis indicates (Figure S2) that the printed monolith closely matches the inner dimensions of the sleeve and no continuous interfacial voids were observed. The monolith was inserted into a sleeve specified for capillaries with outer diameters of 275–315 µm, while the printed structures exhibited only approximately 4 % shrinkage relative to the CAD dimensions. Furthermore, insertion of fused-silica capillaries (365 µm o.d.) locally expands the FEP sleeve, generating a mechanically tight and leak-free connection. These observations suggest that any wall-channeling contribution is likely small compared to the extra-column dispersion originating from the fluidic interfaces and detector volume.

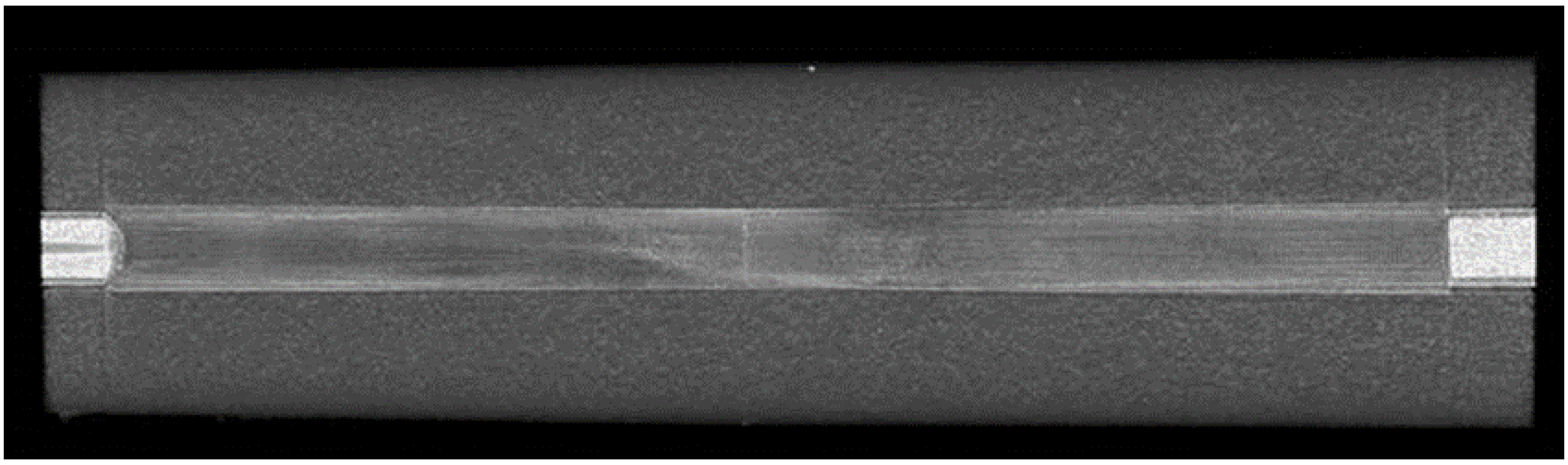

Figure S2. Nano-CT cross-sectional image of microprinted polymer monolith incorporated into FEP sleeve used for chromatographic experiments.

Comment on Chromatographic Performance and Technical Limitations of the System Presented

The chromatographic experiments presented in this work demonstrate the feasibility of using two-photon polymerization to fabricate hierarchically structured polymer monoliths for miniaturized reversed-phase liquid chromatography. The performance observed in this proof-

of-concept study should not be interpreted as representing the intrinsic or theoretical performance limits of TPMS-based stationary phases.

The mechanical stability of the Schoen gyroid used in our study enabled safe handling of the monolith during its insertion into the sleeve. However, numerical studies suggested that the transport and chromatographic behavior of TPMS structures depend strongly on geometry, porosity, and characteristic length scale.[4,5] Therefore, for a more comprehensive evaluation of chromatographic performance, alternative TPMS structures will be considered in subsequent studies.

The present monolith was designed with an approximate porosity of 70 % to ensure low hydraulic resistance and enable integration into a miniaturized LC system. Literature reports suggest that porosities in this range can provide high permeability, although trade-offs with axial dispersion and mass transport are generally expected.[4]

Finally, as described in the manuscript the observed peak broadening is influenced not only by the stationary phase but also by extra-column dispersion. The results should therefore be interpreted as a proof of concept demonstrating fabrication, functionalization, and chromatographic operation of 2PP-printed TPMS monoliths, with performance reflecting combined effects of geometry, fabrication constraints, and system dispersion rather than intrinsic TPMS limits.

References

(1) Hussain, I.; Al-Ketan, O.; Renda, F.; Malvezzi, M.; Prattichizzo, D.; Seneviratne, L.; Abu Al-Rub, R. K.; Gan, D. Design and prototyping soft–rigid tendon-driven modular grippers using interpenetrating phase composites materials. *Int. J. Robot. Res.* **2020**, *39*, 1635–1646.

(2) Toulouse, A.; Thiele, S.; Hirzel, K.; Schmid, M.; Weber, K.; Zyrianova, M.; Giessen, H. Herkommer, A. M.; Heymann, M. High resolution femtosecond direct laser writing with wrapped lens. *Opt. Mater. Express* **2022**, *12*, 3801–3809.

(3) Matheuse, F.; Vanmol, K.; van Erps, J.; de Malsche, W.; Ottevaere, H.; Desmet, G. On the potential use of two-photon polymerization to 3D print chromatographic packed bed supports. *J. Chromat. A* **2022**, *1663*, 462763.

(4) Dolamore, F.; Houlton, B.; Fee, C. J.; Watson, M. J.; Holland, D. J. A numerical investigation of the hydrodynamic dispersion in triply periodic chromatographic stationary phases. *J. Chromat. A* **2022**, *1685*, 463637.

(5) Lauriola, C.; Venditti, C.; Desmet, G.; Adrover, A. Dispersion properties of triply periodic minimal surface stationary phases for LC: The case of superficial adsorption. *J. Chromat. A* **2025**, *1743*, 465676.

(6) Courtois, J.; Byström, E.; Irgum, K. Novel monolithic materials using poly(ethylene glycol) as porogen for protein separation. *Polymer* **2006**, *47*, 2603–2611.

(7) Li, M.; Zhang, H.; Zhong, W.; Ou, J.; He, J.; Wu, Q. Preparation and chromatographic properties of ordered macroporous polymer monoliths by colloidal crystal templating method. *Polym. Bull.* **2025**, *82*, 69–85.

(8) Svec, F. Porous polymer monoliths: Amazingly wide variety of techniques enabling their preparation. *J. Chromat. A* **2010**, *1217*, 902–924.

(9) Simon, U.; Dimartino, S. Direct printing of monolithic ion exchange adsorbers. *J. Chromat. A* **2019**, *1587,* 119–128.

(10) Conti, M.; Symington, J. A.; Pullen, J. R.; Mravljak, R.; Podgornik, A.; Dimartino, S. Porous platform inks for fast and high-resolution 3D printing of stationary phases for downstream processing. *Adv. Mater. Technol.* **2023**, *8,* 2300801.
